# Context changes judgments of liking and predictability for melodies

**DOI:** 10.3389/fpsyg.2023.1175682

**Published:** 2023-11-15

**Authors:** Alexander W. Albury, Roberta Bianco, Benjamin P. Gold, Virginia B. Penhune

**Affiliations:** ^1^Department of Psychology, Concordia University, Montreal, QC, Canada; ^2^International Laboratory for Brain, Music and Sound Research (BRAMS) and Center for Research in Brain, Language and Music (CRBLM), Montreal, QC, Canada; ^3^Neuroscience of Perception and Action Laboratory, Italian Institute of Technology, Rome, Italy; ^4^Department of Electrical and Computer Engineering, Vanderbilt University, Nashville, TN, United States

**Keywords:** musical prediction, reward, expectation, melodic pleasure, predictive coding, contrast, comparison

## Abstract

Predictability plays an important role in the experience of musical pleasure. By leveraging expectations, music induces pleasure through tension and surprise. However, musical predictions draw on both prior knowledge and immediate context. Similarly, musical pleasure, which has been shown to depend on predictability, may also vary relative to the individual and context. Although research has demonstrated the influence of both long-term knowledge and stimulus features in influencing expectations, it is unclear how perceptions of a melody are influenced by comparisons to other music pieces heard in the same context. To examine the effects of context we compared how listeners’ judgments of two distinct sets of stimuli differed when they were presented alone or in combination. Stimuli were excerpts from a repertoire of Western music and a set of experimenter created melodies. Separate groups of participants rated liking and predictability for each set of stimuli alone and in combination. We found that when heard together, the Repertoire stimuli were more liked and rated as less predictable than if they were heard alone, with the opposite pattern being observed for the Experimental stimuli. This effect was driven by a change in ratings between the Alone and Combined conditions for each stimulus set. These findings demonstrate a context-based shift of predictability ratings and derived pleasure, suggesting that judgments stem not only from the physical properties of the stimulus, but also vary relative to other options available in the immediate context.

## 1. Introduction

The experience of music is a complex process that involves mechanisms of reward (Salimpoor et al., 2009, 2013, 2015; for review see Zatorre and Salimpoor, 2013; Cheung et al., 2019; Belfi and Loui, 2020; Mas-Herrero et al., 2021), as well as predictive processes about unfolding music that rely on regularities learned through prior listening experience (Pearce and Wiggins, 2012; Bianco et al., 2016; Hansen et al., 2016; for review see Koelsch et al., 2019). Many theories posit that musical reward, or rated pleasure, relies on the implicit ability of the brain to make predictions (Vuust et al., 2022), and numerous studies have shown that rated pleasure generally peaks in response to moderate levels of predictability in musical structure (Bianco et al., 2019; Cheung et al., 2019; Gold et al., 2019b; Matthews et al., 2019). This suggests that musical pleasure may arise from an optimally arousing level of surprise generated by a balance of expectations being confirmed or violated (Huron, 2006; Koelsch et al., 2019). Recent evidence has found that during listening, predictions about upcoming events depend not only on long-term context, consisting of the listener’s implicit schematic understanding of musical rules based on lifetime experience (Pearce, 2018), but are also dynamically modulated by the local musical context of the current stimulus (Gold et al., 2019b; Quiroga-Martinez et al., 2019; Bianco et al., 2020). For example, Gold et al. (2019b) found that the uncertainty of a musical sequence systematically affects reported enjoyment, and Quiroga-Martinez et al. (2019), and Bianco et al. (2020) observed this effect at the physiological and neural levels. However, although this evidence supports that the global uncertainty of a musical piece affects perceptions, it is unclear how musical pieces are assessed relative to each other. An open question, then, is how pleasure is modulated by the musical context in which a piece is heard. Here, we investigate how predictions and derived musical pleasure for a stimulus are modulated relative to the presence of other available musical pieces, which we refer to as the reference context.

Musical predictions are believed to stem from implicit statistical learning and probabilistic prediction processes based on the regularities learnt through lifetime exposure to music (i.e., long-term context), as well as through characteristics of the stimulus at hand (i.e., local context; Saffran et al., 1999; Tillmann et al., 2000; Pearce, 2018). Inspired by prominent prediction theories (Friston, 2005, 2010; den Ouden et al., 2012), music perception is thus hypothesized to rely on a combination of long-term schematic and short-term dynamic predictions and error monitoring mechanisms (Vuust et al., 2018, 2022; Koelsch et al., 2019). Under these theories, the brain constantly attempts to minimize the prediction errors arising from the mismatch between predicted and observed events, either by accurately identifying and dismissing erroneous events that do not add value, or by updating the underlying predictive model to account for new information. In this way, listeners rely on a combination of long-term knowledge and stimulus characteristics when making predictions. Accordingly, predictive processes in music have been shown to be sensitive to both long-term or schematic context, such as stylistic expertise (Hansen et al., 2016), cultural background (Pearce, 2018), and newly acquired musical rules (Castellano et al., 1984; Loui and Wessel, 2008; Loui et al., 2010), as well as stimulus-dependent local context such as the predictability or uncertainty of the stimulus (Southwell and Chait, 2018; Gold et al., 2019b; Quiroga-Martinez et al., 2019; Bianco et al., 2020).

The extent to which music conforms to or deviates from the listener’s predictions has been hypothesized to influence emotional arousal and reward responses (Berlyne, 1970; Huron, 2006; Pearce and Wiggins, 2012). In line with this view, physiological responses such as pupil dilation and skin conductance are modulated by music predictability (Steinbeis et al., 2006; Koelsch et al., 2008; Egermann et al., 2013; Bianco et al., 2019, 2020), and an inverted U-shaped curve underpinning the relationship between music enjoyment and predictability indicates that music of intermediate predictability is frequently the most preferred (Berlyne, 1970; Witek et al., 2014; Chmiel and Schubert, 2017; Bianco et al., 2019; Cheung et al., 2019; Gold et al., 2019b; Matthews et al., 2019). These results suggest a link between the predictability of a musical piece and the corresponding pleasure or reward derived from it.

However, musical pleasure is not an absolute construct; the same musical piece can elicit different reactions, even from the same listener. For example, a piece of classical music might be judged very differently if heard after a heavy metal song, and vice versa, and the order of presentation of musical stimuli has been shown to influence liking ratings (Parker et al., 2008). This suggests that experienced pleasure may be shaped by the reference context in which music is heard. Accordingly, studies of reward and hedonic contrast demonstrate that the same stimuli can induce different levels of reward under different circumstances. Research in non-human primates has shown that encoding of reward value can be affected by preference such that dopamine neurons discriminate for animals’ relative preference among the available rewards, irrespective of physical stimulus characteristics (Tremblay and Schultz, 1999). Similarly, neurobiological reward has been shown to be influenced by comparisons to other rewarding stimuli in both rats and humans (Lopez-Persem et al., 2016; Webber et al., 2016), and there is evidence that dopamine response to rewarding stimuli is sensitive to motivation and context (see Schultz, 2013 for a review). The source of this reward is believed to rely on general adaptive mechanisms used to motivate behavior and related to other rewarding stimuli (Skov, 2019; Mas-Herrero et al., 2021). There is also behavioral evidence in humans indicating that context and expectations can affect aesthetic evaluation (Zellner et al., 2003; Parker et al., 2008; Belfi et al., 2021; Kolbeinsson et al., 2022; Shank et al., 2022) and neuroimaging evidence which shows that expertise can affect activity in reward areas when judging aesthetic stimuli (Kirk et al., 2009). Taken together, this evidence suggests that experienced pleasure for a stimulus is malleable and can vary relative to the broader reference context in which that stimulus is experienced.

Given the link between musical reward and music predictability, it stands to reason that both listeners’ experiences of pleasure and predictability may be shaped by more than just the long-term schematic information and local stimulus features but also by the relative context in which the music is heard. Here, we test the extent to which judgments of predictability and pleasure shift based on the reference context in which a piece of music is heard. In a series of online studies, participants listened to two structurally distinct sets of musical stimuli, either alone or in combination, and rated how much they liked them and how predictable they were. The two stimulus sets consisted of excerpts from the repertoire of Western music (*Repertoire*), and experimenter created melodies (*Experimental*). They were chosen because they were both expected to induce an inverted U-shaped relationship between liking and predictability when presented alone (see Bianco et al., 2019; Gold et al., 2019b), yet the two sets were stylistically different enough to represent an alternative to each other when presented in combination. We then compared listeners’ ratings across contexts and looked for changes in the predicted inverted U-shaped relationship between predictability and liking. Finally, we examined the relationship between liking ratings and estimates of musical predictability derived from a computational model of statistical learning and probabilistic expectations (IDyOM; Pearce, 2005). We hypothesized that participant ratings would change significantly across contexts, indicating that predictability and reward in music are experienced as relative, rather than absolute constructs, and are sensitive to the musical environment in which a piece of music is heard. For example, if one set of stimuli is preferable to the other, then hearing them in combination may widen this discrepancy. Alternatively, hearing melodies in a combined context could adjust perceptions of only one set of stimuli, bringing them closer to the other set.

## 2. Materials and methods

### 2.1. Stimuli

Stimuli consisted of 3 sets: (1) Repertoire set: this was comprised of 24 monophonic melodies adapted from Gold et al. (2019b). The Repertoire stimuli consisted of monophonic excerpts from Western music sourced from MIDI databases such as https://www.classicalarchives.com/midi.html. These melodies contained a range of note durations, note densities, and tempi, with an average duration of 8.8 s (SD = 0.74) and bpm ranging from 110 to 140. (2) Experimental set: 24 experimenter-composed, monophonic melodies created for a previous experiment (Bianco et al., 2019). The Experimental stimuli were each 13 notes long and written in 3/4 time, with the first 12 notes being quarter-notes and the final note a dotted half-note. The Experimental melodies were played at a tempo of 140 bpm and all had the same duration of 6.4 s. Both the Repertoire and Experimental stimuli were created using *Musescore* with a simulated Grand Piano tone and identical volume and were exported to WAV files for Experimental presentation. (3) Combined set: this set consisted of both the Repertoire and Experimental stimuli presented in random order.

### 2.2. Task and conditions

Three different groups of participants listened to and rated the stimulus sets either alone (Repertoire Alone; Experimental Alone) or in combination (Combined). In each context condition participants heard the melodies one at a time and provided liking and predictability ratings on a Likert scale from 1 (Not at all) to 7 (A lot/Very). For the liking ratings, participants were asked, “How much did you like this melody?,” and for predictability participants were asked, “How predictable was this melody?” Participants were asked to try to make use of the full rating scale. Liking and predictability were rated in separate blocks to minimize the potential for ratings of one measure to affect ratings of the other. The order of stimuli in each set, and whether liking or predictability was rated first were randomized across participants in each condition.

### 2.3. Sequence predictability modeling

In an attempt to obtain an objective measure of statistical predictability as an alternative to listener’s ratings of predictability, the statistical probability of each note in each of the stimuli was calculated using an information theoretic model of music (IDyOM; Pearce, 2005). IDyOM quantifies the probability of every event in a sequence by assigning a value of information content (IC) which is the negative logarithm of the event’s probability. Accordingly, low information content represents events that are highly predictable while higher information content represents events that are unpredictable. IDyOM can generate probability estimates based on both the local context of the current stimulus and simulated long-term knowledge from prior training on a corpus of Western music. The IDyOM model used was trained on 152 Canadian folk songs (Creighton, 1966), 566 German folk songs from the Essen folk song collection (Schaffrath, 1992), and 185 Bach chorale melodies (Riemenschneider, 1941). In this study, we used the *both+* IDyOM configuration which estimates probability based on both long-term and short-term information, but also progressively updates the model as it processes each stimulus. Information content was calculated for each of the three stimulus sets (Repertoire, Experimental, and Combined) independently. We randomized the processing order of the stimuli in the Combined context to mimic the listening experience of participants. Because our focus was on perceptions of each stimulus, we calculated the mean information content for each stimulus by averaging the information content of all notes in that melody. The mean information content for each of the stimulus conditions was 6.1 for the Repertoire stimuli set (min = 3.7, max = 10.8), 4.5 for the Experimental stimuli set (min = 2.8, max = 8.1), and 5.3 in the Combined context (min = 2.8, max = 10.8). Mean IC was significantly greater in the Repertoire stimuli set both when comparing the two Alone contexts (*t* = 3.28, *p* = 0.002) and between the stimuli sets in the Combined context (*t* = −3.21, *p* = 0.002). The average information content of each melody was intended as a statistical measure of predictability to complement listener’s ratings of predictability.

### 2.4. Participants

Three groups of participants were recruited using Prolific.com.^1^ Participants were prescreened to include people from the United States and Canada who indicated in their Prolific profile that they did not play a musical instrument. There were 78 participants who heard the Experimental melodies alone (58 F; Age: *M* = 29.0), 74 who heard the Repertoire melodies alone (55 F; Age: *M* = 26.1), and 83 who heard both sets of stimuli in the Combined context (59 F; Age: *M* = 25.7; Table 1). Participants in the Experimental stimuli group had an average of 3.5 years of music experience. Participants in the Repertoire stimuli group had an average of 3.1 years of music experience. Participants in the Combined context had an average of 3.7 years of music experience. One-way ANOVAs indicated that the groups did not differ in age [*F*(2, 232) = 2.01, *p* = 0.17], or years of music experience [*F*(2, 232) = 0.22, *p* = 0.81], and a Pearson Chi-squared test indicated there were no significant differences in gender distribution [χ^2^(2) = 0.18, *p* = 0.91]. The protocol was approved by the Concordia University Human Research Ethics Committee. Participants provided written informed consent in accordance with the Declaration of Helsinki and were compensated for their time.

**TABLE 1:** Sample characteristics of context groups.

	Repertoire Alone (SD)	Experimental Alone (SD)	Combined (SD)
Age	26.1 (8.27)	29.0 (11.2)	25.7 (8.89)
Gender (F)	55	58	59
Music experience	3.1 (4.30)	3.5 (7.46)	3.7 (5.21)
Total (*n*)	74	78	83

### 2.5. Procedure

Participants received a recruitment link through *Prolific* to the task which was hosted on *Pavlovia.org* and built using *PsychoPy* (Peirce et al., 2019). Data were collected in separate batches for each context condition and participants who had participated in a previous study were not permitted to sign up for another. After completing a consent form, participants were asked demographic questions relating to their music experience.

### 2.6. Analysis

Data were analyzed using R version 4.0.5 (R Core Team, 2021) and Rstudio 2021.09 (RStudio Team, 2021). The difference in ratings between the Alone and Combined conditions for each melody set was evaluated using a one-sample *t*-test to test difference from zero. All other analyses used linear mixed effects models implemented by the *lme4* package (Bates et al., 2015). For all mixed effects analyses, models included random intercepts by participants and stimuli.

Statistical significance of main effects was evaluated by likelihood-ratio tests in which the model was progressively built by adding main effects, and each model was compared to the previous model to determine the significance of the added effect. Likelihood estimation was conducted using the *anova* function in the *stats* package. Follow-up contrasts were conducted using the *emmeans* package (Lenth, 2021). Contrasts for liking and predictability ratings across groups compared means between stimuli sets (Repertoire/Experimental) and contexts (Alone/Combined). Contrasts for the relationships between liking and predictability, and liking and mean information content compared the quadratic coefficients across stimuli sets and contexts estimated using the *emtrends* function from *emmeans*. All contrasts are reported at the level at which the relevant variable was added to the model. Degrees of freedom approximation was conducted using the Kenwood-Roger method, and *p*-values were adjusted for multiple comparisons using the Tukey method for the quadratic coefficients, and the multivariate *t* method for all other contrasts.

**FIGURE 1:**
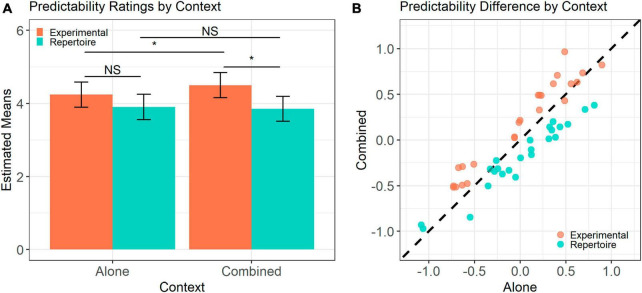
Predictability ratings by context. **(A)** Estimated marginal means for each stimulus set in each context. Error bars represent confidence intervals of estimates. **(B)** Mean participant standardized predictability rating for each melody in the Alone and Combined contexts. Diagonal represents a difference of zero between contexts. Difference from zero is significant at *p* < 0.001 for both stimuli sets. * Indicates *p* < 0.05.

**FIGURE 2:**
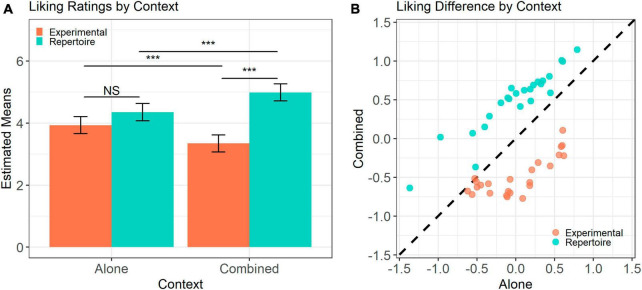
Liking ratings by context. **(A)** Estimated marginal means for each stimulus set in each context. Error bars represent confidence intervals of estimates. **(B)** Mean participant standardized liking rating for each melody in the Alone and Combined contexts. Diagonal represents a difference of zero between contexts. Difference from zero is significant at *p* < 0.001 for both stimuli sets. *** Indicates *p* < 0.001.

**FIGURE 3:**
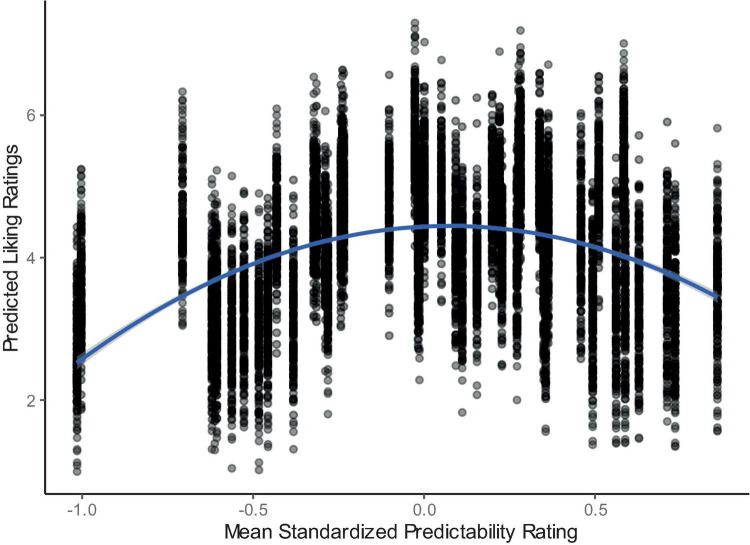
Liking ratings predicted by the linear mixed effects model at each level of mean predictability ratings. Each point represents the predicted liking rating for each participant for each stimulus at the mean standardized predictability rating of that stimulus. Blue line represents a smoothing line on the predicted values using a quadratic term and generalized additive model.

**FIGURE 4:**
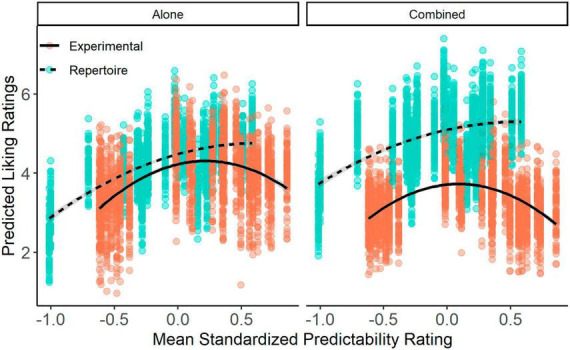
Relationship between liking and predictability ratings. Liking ratings predicted by the linear mixed effects model at each level of mean predictability ratings. Each point represents the predicted liking rating for each participant for each stimulus at the mean standardized predictability rating of that stimulus. Data is divided by context (Left: Alone; Right: Combined) and stimulus set (Blue: Repertoire; Orange: Experimental). Lines represent smoothing lines on the predicted values using a quadratic term and generalized additive model.

**FIGURE 5:**
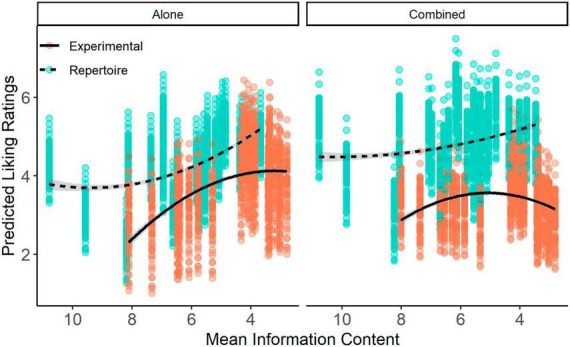
Predicted liking ratings from the mixed effects model plotted against mean information content. Each point represents the predicted liking rating for each participant for each stimulus at the mean information content (IC) of that stimulus. The *x*-axis is flipped to facilitate comparison with predictability (i.e., lower IC = more predictable). Data is divided by context (Left: Alone; Right: Combined) and stimulus set (Blue: Repertoire; Orange: Experimental). Lines represent smoothing lines on the predicted values using a quadratic term and generalized additive model.

While all analyses were conducted using the untransformed data with by participant random effects, participant standardized liking and predictability ratings are used for visualizations of the rating change by context (Figures 1B, 2B). In order to present the relationships captured by the linear mixed effects models, the visualizations of the liking-predictability (Figures 3, 4) and liking-IC (Figure 5) relationships display values of liking ratings predicted by the model at each level of mean predictability or mean IC, respectively.

## 3. Results

### 3.1. Predictability

Figure 1A illustrates the results of the estimated predictability ratings in each context condition derived from the mixed effects model. We found a significant main effect of stimulus set [Repertoire: *M* = 3.88, SD = 1.71; Experimental: *M* = 4.38, SD = 1.72; *b* = −0.60, χ^2^(1) = 6.81, *p* = 0.009] and an interaction between stimulus set and context [χ^2^(1) = 7.56, *p* = 0.006]. Follow-up contrasts indicate that that whilst there were no significant differences between the predictability ratings of Repertoire (*M* = 3.90) and Experimental (*M* = 4.24) melodies when presented separately [*t*(70.2) = −1.39, *p* = 0.438], Experimental melodies (*M* = 4.50) were rated significantly higher than Repertoire melodies (*M* = 3.85) in the Combined context [*t*(51.8) = −2.86, *p* = 0.021]. This effect was driven by a significant increase in the predictability ratings of Experimental stimuli from the Alone (*M* = 4.24) to Combined (*M* = 4.50) context [*t*(257.6) = 2.59, *p* = 0.035], suggesting that these simple melodies were perceived as even simpler when presented amidst the more complex Repertoire melodies. The shift in predictability ratings of the Repertoire stimuli from the Alone (*M* = 3.90) to the Combined context (*M* = 3.85) was not significant [*t*(257.6) = −0.51, *p* = 0.943], although the Repertoire stimuli were rated as less predictable in the Combined context. To confirm the observed shift in ratings between contexts, we compared the difference between ratings to zero (Figure 1B). We conducted one-sample *t*-tests for both the Experimental [*t*(23) = 6.75, *p* < 0.001] and Repertoire stimulus sets [*t*(23) = −5.90, *p* < 0.001] which both showed a clear shift in opposite directions such that the Repertoire stimuli were rated as less predictable and Experimental stimuli were rated as more predictable in the Combined context.

### 3.2. Liking

Figure 2A illustrates the results of the liking ratings derived from the mixed effects model. We found a significant main effect of stimulus set [Repertoire: *M* = 4.6, SD = 1.59; Experimental; *M* = 3.6, SD = 1.60; χ^2^(1) = 48.93, *p* < 0.001] and an interaction between stimulus set and context [χ^2^(1) = 96.28, *p* < 0.001]. Follow-up contrasts indicate that whilst there were no significant differences in liking ratings between the stimulus sets in the Alone context [Repertoire: *M* = 4.35; Experimental: *M* = 3.93; *t*(93.4) = 2.13, *p* = 0.116], in the Combined context, the Repertoire stimuli (*M* = 4.99) were more liked than the Experimental stimuli [*M* = 3.35; *t*(52.8) = 9.70, *p* < 0.001]. The difference in liking ratings between sets in the Combined context was driven by an opposite context-driven shift in both the Experimental and the Repertoire melodies. The Repertoire stimuli were significantly more liked in the Combined context (*M* = 4.99) than in the Alone context [*M* = 4.35; *t*(252.2) = 6.01, *p* < 0.001]. In contrast, the Experimental stimuli were significantly less liked in the Combined context (*M* = 3.35) compared to the Alone context [*M* = 3.93; *t*(252.5) = −5.60, *p* < 0.001]. Figure 2B illustrates the difference in liking ratings based on context. Confirmatory one-sample *t*-tests showed that the difference between the mean standardized liking rating of each melody in the Combined and Alone contexts was significantly different from zero for both the Experimental [*t*(23) = −8.56, *p* < 0.001] and Repertoire stimulus sets [*t*(23) = 12.75, *p* < 0.001]. These results suggest that the pleasure derived from a piece of music depends on the reference context in which the piece is heard.

### 3.3. Predictability and liking relationship

To test the relationship between liking and predictability we compared participant ratings in a mixed effect model with liking as the dependent variable. Because of previous evidence for an inverted U-shaped, or quadratic, relationship between liking and predictability, we tested whether this relationship was present across contexts. First, we compared both linear and quadratic fits for all stimuli and all contexts, and found that, while the linear relationship was significant [χ^2^(1) = 17.21, *p* < 0.001] a quadratic relationship fit the data significantly better than the linear relationship [χ^2^(1) = 15.71, *p* < 0.001; Figure 3].

There were also significant interactions between predictability and context [χ^2^(2) = 75.78, *p* < 0.001] and predictability and stimulus set [χ^2^(2) = 35.48, *p* < 0.001], as well as a three-way interaction between context, stimulus set, and predictability in their effect on liking ratings [χ^2^(2) = 17.02, *p* < 0.001]. Because the quadratic model provided an overall better fit and because previous evidence predicts a quadratic relationship between liking and predictability (Bianco et al., 2019; Gold et al., 2019b; Matthews et al., 2019), we conducted follow-up contrasts comparing the quadratic coefficient across different contexts and stimulus sets (Figure 4). The stimulus sets showed similar quadratic curves in the Alone context [*t*(7,603) = −2.14, *p* = 0.387], but in the Combined context the Experimental set exhibited a more distinct inverted U-shaped relationship than the Repertoire set, evidenced by a larger quadratic coefficient [*t*(432) = −6.077, *p* < 0.001]. However, it is worth noting that the observed change, though significant, represents a small difference in the quadratic curve between the stimulus sets in the Combined context.

Comparisons within stimulus sets yielded no significant context-driven shift in the quadratic curve. There was no significant difference in the size of the quadratic coefficients between the Alone and Combined contexts for the Experimental stimuli {Alone: *b*(7,609) = −0.04, 95% CI [−0.060, −0.019]; Combined: *b*(7,474) = −0.06, 95% CI [−0.080, −0.036]; *t*(7,553) = 1.23, *p* = 0.92}, or for the Repertoire stimuli {Alone: *b*(7,589) = −0.01, 95% CI [−0.029, 0.013]; Combined: *b*(7,458) = 0.03, 95% CI [0.013, 0.055]; *t*(7,526) = −2.84, *p* = 0.09}. This suggests that the relationship between liking and predictability may be more robust to context than the individual ratings themselves.

### 3.4. Information content and liking

To test the relationship between information content estimates from IDyOM and participant predictability ratings, a mixed effects model containing only these variables and random intercepts by participant was used. As expected, there was a significant linear relationship between mean information content and predictability, with predictability ratings decreasing as information content increased [*b* = −0.32, χ^2^(1) = 36.07, *p* < 0.001].

Similar to the effects of predictability on liking, we assessed the effects of mean information content on liking. There was no significant linear [χ^2^(1) = 0.95, *p* = 0.330] or quadratic [χ^2^(1) = 1.35, *p* = 0.244] relationship between mean information content and liking ratings. There was a significant interaction between mean information content and context on liking [χ^2^(2) = 115.02, *p* < 0.001]. The relationship between stimulus set and mean information content on liking was not significant [χ^2^(2) = 1.34, *p* = 0.511] but there was a significant three-way interaction between stimuli, context, and mean information content [χ^2^(2) = 8.39, *p* = 0.015], suggesting that the stimuli may display varying liking-predictability relationships across contexts.

However, similar to the results of the participant liking and predictability ratings, follow-up contrasts (Figure 5) showed minimal differences in the relationship between liking and information content across stimuli and contexts. There was no significant difference between quadratic coefficients in the Experimental Alone {*b*(72) = 0.07, 95% CI [−0.117, 0.249]} and Experimental Combined {*b*(72) = 0.08, 95% CI [−0.117, 0.268]} conditions [*t*(6,792) = −0.009, *p* = 0.999], with both displaying similar quadratic curves. There was also no significant difference between the Repertoire Alone {*b*(204) = 0.05, 95% CI [−0.014, 0.116]} and Repertoire Combined {*b*(192) = 0.04, 95% CI [−0.024, 0.097]} conditions [*t*(7,394) = 0.01, *p* = 0.911], with both appearing to show a linear relationship, with liking decreasing as information content increased.

In the comparison between stimulus sets, there was no significant difference in quadratic coefficients between the Experimental Alone and Repertoire Alone conditions [*t*(77) = 0.02, *p* = 0.999], or between the Experimental Combined and Repertoire Combined conditions [*t*(75) = 0.03, *p* = 0.999]. The lack of differences between quadratic coefficients despite the significant three-way interaction observed between liking, context, and stimuli is driven by minor changes in the linear relationships of the conditions. Although our research questions focus on quadratic relationships, *post hoc* analysis of the linear slopes revealed that there were minor differences in the linear slopes between the Alone and Combined contexts. However, the confidence intervals of these slopes also crossed zero, and as reported above there was no main effect of either a linear or quadratic relationship. Altogether, these results suggest that the relationship between liking ratings and information content do not vary significantly based on context.

## 4. Discussion

We investigated how ratings of liking and predictability for Repertoire and Experimental melodies changed depending on whether they were heard alone or in combination. We found that while there was no difference between ratings of the melodies in their respective Alone conditions, the Repertoire stimuli were rated as less predictable and more liked than the Experimental stimuli when they were heard together. We also found that, relative to themselves, the Experimental stimuli were more liked when heard alone, and the Repertoire stimuli were more liked when heard in the Combined context. These results indicate that perceptions of predictability and liking for a piece of music are modulated by the context of other available melodies in which that piece is heard. We also examined the inverted U-shaped relationship between liking and predictability ratings, and between liking ratings and information content derived from an information theoretic model of music (IDyOM). We observed an inverted U-shaped relationship between liking rating and predictability ratings but not between liking ratings and information content. Both of these relationships showed little variation, with both predictability ratings and information content appearing to remain consistent in their relationship to liking regardless of context.

The change in liking ratings in the alone and combined contexts demonstrate that the same stimuli can elicit different responses in different circumstances. This is consistent with other research that indicates that the reward and pleasure associated with a stimulus are context dependent, namely relative to the set of alternatives in which the stimulus is embedded (Tremblay and Schultz, 1999) and the expectations of the individual (Belfi et al., 2021; Kolbeinsson et al., 2022; Shank et al., 2022). By changing the context in which our stimuli were presented, we aimed to induce a shift in the reference context used by participants for evaluating the stimuli. It’s likely that participants began with a baseline set of expectations and judgments and then adjusted these based on new information.

Our results mirror studies of contrast effects for aesthetic and social stimuli. For example, Zellner et al. (2003) employed a similar design in which one group of participants rated full-strength fruit juices and diluted fruit-juices while another group tasted only the diluted juices. Participants who only tasted the diluted juices gave them higher ratings than those in the combined group. In music, Parker et al. (2008) compared participant reactions to melodies that had been designed to be “good” or “bad” and found that pleasant melodies were rated higher when heard after unpleasant melodies, and unpleasant melodies were rated lower if heard after pleasant melodies. Our results further support the effect of hedonic contrast for musical stimuli. Distinctions between the stimulus sets appeared despite participants in the Combined condition not being explicitly informed that there were two distinct stimulus groups. While participants may have categorized the stimuli groups, previously observed effects of categorization in hedonic contrast are mixed. Parker et al. (2008) found no effects of categories (i.e., labeling stimulus sets as being from unique cultures) in their study. Additionally, when they are present, categorization effects are theorized to reduce or completely eliminate hedonic contrast effects (Zellner et al., 2003), suggesting that there were minimal categorization effects in the current study.

Music reward is closely linked with musical expectations (Gold et al., 2019a,b; Bianco et al., 2020); therefore, it is likely that musical pleasure and predictability are malleable in the same way. This was supported by the observation that, as with liking ratings, predictability ratings also exhibited a context-based shift. Expectations are based on information sampled from our environment which means that our predictions are constantly being updated (Koelsch et al., 2019). Previous research has shown that musical predictions are shaped by prior exposure to music. For example, in a study comparing listener ratings of predictability to probability estimates from a computational model of music, Hansen et al. (2016) found that jazz musicians’ predictions better matched those from a model trained on bebop music than did those of classical musicians or non-musicians. Similarly, songs that are statistically predictable based on Western pitch probabilities are less predictable when evaluated using pitch probabilities from Chinese music, and vice versa, suggesting that listeners from these cultures have differing expectations of musical events (Pearce, 2018; Klarlund et al., 2023).

However, in addition to relying on long-term priors, people are capable of quickly updating their expectations of music based on new information. For example, Hansen et al. (2016) reported that when listening to bebop music, ratings from listeners with no bebop experience still fit well with estimates from a model trained on bebop music, suggesting that listeners were not applying irrelevant statistical knowledge to the jazz music but instead dynamically adapting their predictions to the context. Similarly, Western participants do not apply Western pitch expectations when listening to Indian music, as shown by similar performance on a probe tone task with Indian melodies (Castellano et al., 1984; but see Krumhansl et al., 2000). Further, research has shown that people are capable of learning a new musical pitch system in less than an hour (Loui and Wessel, 2008; Loui et al., 2010). There are also similar accounts in the statistical learning literature that support rapid learning of artificial grammar for language (Aslin et al., 1998) and tones (Saffran et al., 1999). Overall, whilst there is evidence that predictions are sensitive to experience and can be quickly adapted to new regularities in the stimuli, the shift in participant predictability ratings observed in our study indicates that our perceptions of predictability are also affected by the relative predictability estimated across the items presented in a reference context.

Previous studies have shown an inverted U-shaped relationship between liking and predictability (Witek et al., 2014; Bianco et al., 2019; Gold et al., 2019b; Matthews et al., 2019). We found little change in this relationship across contexts, suggesting that although overall ratings of liking and predictability may shift, the relationship between them may be more robust to changes in stimulus context. This finding further supports the stability of the inverted U-shaped relationship between liking and predictability that has been observed across multiple study paradigms, populations, and stimuli (see Chmiel and Schubert, 2017 for a review), which in themselves can be viewed as different musical contexts. The consistency of this relationship in comparison to the change in ratings is noteworthy because it implies that listeners’ predictions and experienced pleasure change in tandem.

In addition to listener-rated predictability, we also examined the relationship between liking ratings and probability estimates from a computational model of melodic predictability. We found that mean information content from the IDyOM model significantly explained participant predictability ratings, with rated predictability decreasing as information content increased. This suggests some comparability between these two metrics. Similar to the relationship between participant liking and predictability ratings, there was little variability in the relationship between liking and information content across stimuli and contexts. In all conditions the relationship between liking and information content appears to be linear, with liking decreasing as melodies become more complex. This is similar to the relationship between liking and information content observed in Bianco et al. (2019) which used similar stimuli as the Experimental stimuli. The similar effects of subjective predictability ratings and information content on liking suggest that IDyOM captures some of the same regularities used by human listeners in their judgements of predictability.

However, we did not observe an inverted U-shaped relationship between liking and information content as we did with participant predictability ratings, in that there were no significant main effects of either linear or quadratic information content on liking. A potential explanation for this could be that despite using unique IDyOM models for each context, the predictions generated by IDyOM in this experiment were not as sensitive to relations across stimuli as the human listeners appear to be, perhaps because human listeners give more weight to stimulus boundaries. It is also worth noting that IDyOM estimates probabilities on a note-by-note basis, but in our analyses, mean information content of the melody was used as a predictor. It is possible that averaging information content across all notes in a melody is not a wholly accurate representation of its predictability.

Although neither quadratic or linear relationships significantly explained the relationship between mean IC and liking, the relationships appear to be more linear rather than following an inverted-U. This apparent linear trend in IC, along with the linear relationship between liking and predictability ratings in the Repertoire stimuli are worth interpreting in light of the varied findings related to the inverted-U in musical pleasure (see Chmiel and Schubert, 2017 for review). In their review, Chmiel and Schubert (2017) report several studies in which linear relationships were observed instead of an inverted-U. They explain this by noting that the inverted-U curve in aesthetic appreciation has been theorized to consist of separate segments. It’s possible that the Repertoire stimuli did not contain a wide enough range of predictability and so only represent a segment of the inverted-U relationship.

Although our findings indicate that perceptions of musical pleasure and predictability are susceptible to change based on a reference context, it remains unclear what circumstances or musical characteristics prompt this change. Under theories of predictive coding, our cognitive models are constantly updating to produce more accurate predictions. To do this, these internal predictive processes must be sensitive to short-term information while also retaining a more global, long-term set of predictions. Cognitive models of music prediction are hypothesized to be robust to minor deviations such as isolated wrong notes, with such events receiving little weight at higher levels of processing, and thus having little effect on the overall predictive model (Koelsch et al., 2019; Quiroga-Martinez et al., 2021). However, as discussed previously, these models are sensitive to experience (Hansen et al., 2016; Pearce, 2018; Klarlund et al., 2023) and so listeners must reconcile both short-term and long-term knowledge when generating predictions. Here, we show that beyond long-term schematic information and local stimulus statistics, changes in predictability judgments are also shaped by the relative context in which the music is heard. It remains unclear how much listeners weight information across this continuum (i.e., from long-term knowledge, to reference context, to local statistics) when making predictions about music, as well as what is the exact time course by which they incorporate short-term information into a more long-term model of musical expectations. Alternatively, listeners may entirely compartmentalize musical knowledge instead of relying on a more general foundation of musical patterns and probabilities. There may be contexts in which listeners rely almost exclusively on local and reference information such as when they are presented with stimuli that do not fit well with their existing musical knowledge.

Altogether, our results indicate that perceptions of music are formed relative to the set of available options within a reference context, and that the same piece of music can be judged in different ways, not only based on its statistical properties but also depending on the context in which it is heard. When presented together, Repertoire stimuli were judged as more likeable and less predictable than Experimental stimuli, which were less liked and perceived as more predictable. However, the relationship between liking and predictability appears relatively unaffected by context. We interpret these findings in the view of musical expectations intertwined with experience of pleasure and based on integration of contextual information at different time scales.

## Data availability statement

The raw data supporting the conclusions of this article will be made available by the authors, without undue reservation.

## Ethics statement

The studies involving humans were approved by the Concordia University Office of Research, Research Ethics Unit. The studies were conducted in accordance with the local legislation and institutional requirements. The participants provided their written informed consent to participate in this study.

## Author contributions

AA collected data, conducted analyses, and wrote the manuscript. All authors contributed to the conception and design of the study, manuscript revision, read, and approved the submitted version.

## Footnotes

1
https://www.prolific.com/
